# Coastal ocean and shelf-sea biogeochemical cycling of trace elements and isotopes: lessons learned from GEOTRACES

**DOI:** 10.1098/rsta.2016.0076

**Published:** 2016-11-28

**Authors:** Matthew A. Charette, Phoebe J. Lam, Maeve C. Lohan, Eun Young Kwon, Vanessa Hatje, Catherine Jeandel, Alan M. Shiller, Gregory A. Cutter, Alex Thomas, Philip W. Boyd, William B. Homoky, Angela Milne, Helmuth Thomas, Per S. Andersson, Don Porcelli, Takahiro Tanaka, Walter Geibert, Frank Dehairs, Jordi Garcia-Orellana

**Affiliations:** 1 Department of Marine Chemistry and Geochemistry, Woods Hole Oceanographic Institution, Woods Hole, MA 02543, USA; 2 Department of Ocean Sciences, University of California-Santa Cruz, Santa Cruz, CA 95064, USA; 3 Ocean and Earth Science, National Oceanography Centre, University of Southampton, Southampton SO14 3ZH, UK; 4 Research Institute of Oceanography, Seoul National University, Seoul 151-742, Korea; 5 Centro Interdisciplinar de Energia e Ambiente, Inst. de Química, Universidade Federal da Bahia, Salvador 40170-115, Brazil; 6 University of Toulouse/CNRS/UPS/IRD/CNES, Observatoire Midi-Pyrénées, Toulouse 31400, France; 7 Department of Marine Science, University of Southern Mississippi, Stennis Space Center, MS 39529, USA; 8 Department of Ocean, Earth, and Atmospheric Sciences, Old Dominion University, Norfolk, VA 23529, USA; 9 School of GeoSciences, University of Edinburgh, Edinburgh EH9 3FE, UK; 10 Institute of Marine and Antarctic Studies, University of Tasmania, Hobart, Tasmania 7005, Australia; 11 Department of Earth Sciences, University of Oxford, Oxford OX1 3AN, UK; 12 School of Geography, Earth and Environmental Sciences, Plymouth University, Plymouth PL4 8AA, UK; 13 Department of Oceanography, Dalhousie University, Halifax, Nova Scotia, Canada B3H 4R2; 14 Department of Geosciences, Swedish Museum of Natural History, Stockholm 104 05, Sweden; 15 Atmosphere and Ocean Research Institute, University of Tokyo, Kashiwanoha 5-1-5, Kashiwa Chiba 277-8564, Japan; 16 Marine Geochemistry Department, Alfred Wegener Institute Helmholtz Centre for Polar and Marine Research, Am Handelshafen 12, 27570 Bremerhaven, Germany; 17 Earth System Sciences and Analytical, Environmental and Geo-Chemistry, Vrije Universiteit Brussel, Brussels 1050, Belgium; 18 Physics Department-ICTA, Universitat Autònoma de Barcelona, Barcelona 08193, Spain

**Keywords:** GEOTRACES, trace elements, isotopes, radium, continental shelf

## Abstract

Continental shelves and shelf seas play a central role in the global carbon cycle. However, their importance with respect to trace element and isotope (TEI) inputs to ocean basins is less well understood. Here, we present major findings on shelf TEI biogeochemistry from the GEOTRACES programme as well as a proof of concept for a new method to estimate shelf TEI fluxes. The case studies focus on advances in our understanding of TEI cycling in the Arctic, transformations within a major river estuary (Amazon), shelf sediment micronutrient fluxes and basin-scale estimates of submarine groundwater discharge. The proposed shelf flux tracer is 228-radium (*T*_1/2_ = 5.75 yr), which is continuously supplied to the shelf from coastal aquifers, sediment porewater exchange and rivers. Model-derived shelf ^228^Ra fluxes are combined with TEI/ ^228^Ra ratios to quantify ocean TEI fluxes from the western North Atlantic margin. The results from this new approach agree well with previous estimates for shelf Co, Fe, Mn and Zn inputs and exceed published estimates of atmospheric deposition by factors of approximately 3–23. Lastly, recommendations are made for additional GEOTRACES process studies and coastal margin-focused section cruises that will help refine the model and provide better insight on the mechanisms driving shelf-derived TEI fluxes to the ocean.

This article is part of the themed issue ‘Biological and climatic impacts of ocean trace element chemistry’.

## 1. Introduction

Continental shelves and shelf seas play an important role in modulating the transfer of materials between the land and ocean. As such, quantifying processes occurring within this key interface is essential to our understanding of the biogeochemistry of trace elements and their isotopes (TEIs) in the ocean, a major goal of the GEOTRACES programme (www.geotraces.org). Moreover, the supply and removal of elements in coastal oceans have direct influence on the structure of ocean ecosystems and their productivity. Although coastal oceans comprise only around 7% of the total ocean area, they support 15–20% of total primary productivity and provide 90% of the world's fish yield [1]. As a critical Earth system interface, a large proportion of CO_2_ exchange between the ocean and atmosphere occurs over the shelf, which is thought to be a net sink for both atmospheric and terrestrial carbon [2–4].

In the near shore environment, estuaries are known to be important zones of TEI processing [5]. One classic example is the removal of dissolved iron during estuarine mixing, which has been shown in many cases to vastly diminish the riverine flux of this element to the ocean [6–8]. Similarly, uranium has an active biogeochemistry in estuaries and salt marshes, which generally, yet not exclusively, act as sinks for dissolved U [9–11]. Dissolved organic matter (DOM) and several other trace elements may also be removed, at different rates, along the salinity gradient of estuaries and shelves [8,12–15], while some TEIs like barium and radium are known to be added due to desorption from riverine particles [16–20]. In addition to rivers [21], submarine groundwater discharge (SGD) may represent a large source of TEIs to the coastal ocean [22,23]. Comprising a mixture of meteoric groundwater and seawater circulated through coastal aquifers, SGD has been estimated to exceed river discharge both regionally [24,25] and by a factor of 3–4 on a global basis [26]. Furthermore, SGD has been shown to be an important source of micronutrients (e.g. Fe [27]), contaminants (e.g. Hg [28] and Pb [29]), and TEIs commonly used as palaeo-tracers (e.g. U and Ba [30]).

For some elements, boundary exchange processes involving sedimentary deposits on the continental margins may have substantial or even greater fluxes to the ocean than rivers. Diffusive benthic fluxes can be a major source of dissolved rare earth elements (REE) to the ocean at levels that could explain the missing source observed in recent isotopic modelling studies [31–33], where the REE flux from shelf sediments is larger than other REE sources to the ocean [34]. The sedimentary remobilization of Nd along continental margins, specifically due to sediment dissolution, also illustrates the importance of shelf porewater exchange processes as a source of TEIs to the ocean [31]. Studies at ‘mid-ocean’ shelves, such as the Kerguelen and Crozet Plateaus, showed a substantial role of sedimentary iron release in alleviating Fe limitation and enhancing carbon sequestration in the Southern Ocean [35–37].

The GEOTRACES programme has carried out basin-scale sections to quantify and identify the processes that supply TEIs at ocean boundaries (atmosphere–ocean, sediment–water, ocean crust–overlying water, continent–ocean [38–41]). However, the coastal or shelf-ocean is an interface that requires additional process studies to investigate the key processes impacting on the biogeochemical cycles of TEIs. The identification and quantification of TEI distributions and fluxes along ocean margins are important for a number of reasons, including their sensitivity to changing precipitation and wind patterns, and potential impacts on aquaculture and fisheries. Particularly striking is the extent and rate at which humans have modified the coastal zone worldwide [42], a narrow strip of land within 100 km of the ocean where half of the world's population lives and where three-quarters of all large cities are located [43,44]. The impacts are numerous and include large-scale bottom water anoxia, eutrophication, acidification, overfishing and anthropogenic contaminant inputs. For instance, global budgets of TEIs such as Pb and Hg have already been significantly altered in the ocean as a result of human-induced activities such as acid mine drainage [45,46]. The role of changing sea-ice cover may affect shelf TEI transport rates, and TEI discharges associated with the accelerated melting of large ice sheets have the potential to increase in magnitude over the coming decades to centuries. For the present-day Greenland, the Fe flux may already be on par with the total amount of Fe delivered to the North Atlantic Ocean via dust [47], but the scale of this impact depends on the quantification of fluxes between the coast and open ocean [48].

An understanding of the mechanisms governing the linkages between the terrestrial → shelf → open ocean continuum is crucial [49]. Although some GEOTRACES process studies have focused more in near shelf regions, GEOTRACES sections to date have, by design, focused primarily on open ocean transects. Here, we highlight several examples of where GEOTRACES studies have yielded significant insight on shelf TEI processes, defined as those occurring along ocean margins at water depths less than 200 m. We further propose a new approach for quantifying the shelf flux of TEIs using a radium isotope tracer (^228^Ra) and inverse modelling techniques. Finally, we recommend a series of efforts that are necessary to constrain the exchange processes at coastal–shelf ocean interfaces and to aid in the prediction of fluxes of TEIs from this boundary to the ocean.

## 2. Significant GEOTRACES contributions to our understanding of shelf impacts on trace element and isotope budgets for the open ocean

### (a) The Arctic

The Arctic Ocean is unique among the major ocean basins in having as much as one half of its area taken up by shelves [50]. Further, the basin receives a disproportionate percentage of the world's river discharge (10% [51]). Arctic waters are also highly stratified, with a distinct low-salinity surface mixed layer, a strong halocline, and clear shelf and river inputs. Because of these features, the impact of shelf–basin interactions on TEI distributions is particularly prominent throughout the Arctic Ocean. However, TEI data have been limited due to the logistical difficulties of reaching remote and ice-covered regions. The International Polar Year 2007–2008 provided a launching pad for the GEOTRACES programme, with five cruises in the Arctic region between 2006 and 2009, which led to new insights about important Arctic coastal processes acting on TEI distributions. More recently, in summer 2015 three nations mounted full GEOTRACES Arctic cruises; the results of that coordinated effort are forthcoming.

**Figure 1.**
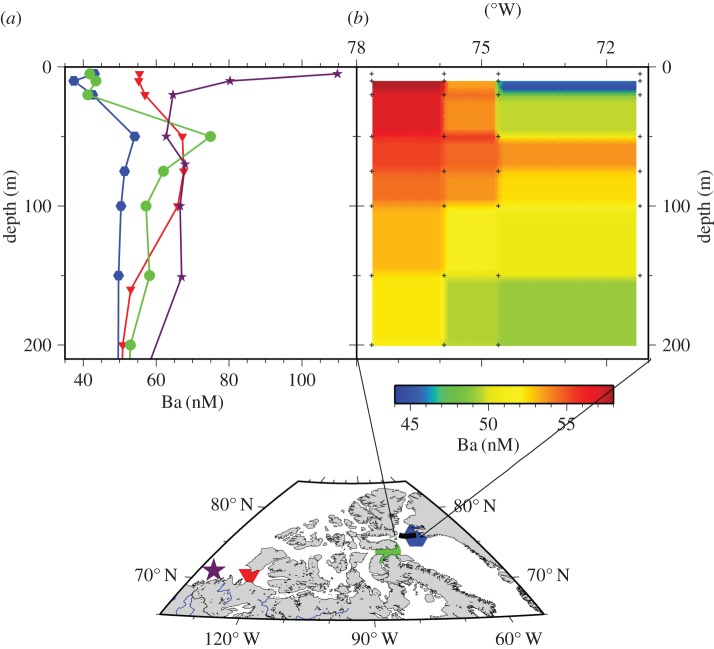
Dissolved Ba concentrations observed in the Canadian Arctic Archipelago during the Canadian CFL-IPY-GEOTRACES programme in 2007–2008. (*a*) Profiles of four selected stations across the archipelago. The easternmost station (hexagons) is under the influence of northward flowing North Atlantic waters, which reveal substantially lower Ba concentrations than waters sampled at stations within the archipelago. The westernmost station (stars) near the Horton River estuary depicts the riverine surface source of Ba. In archipelagic waters (circles), Ba displays a subsurface maximum, which in turn can be used to trace the eastward transport of waters through the archipelago (redrawn after Thomas *et al.* [56]). (*b*) Ba contour section across the head of Baffin Bay, approximately along 76° N, as indicated by the black line in the inserted map in (*a*). The easternmost station is identical with the one shown in (*a*)) (hexagons).

High concentrations of shelf-derived trace metals in surface waters of the central Arctic were reported by Moore [52]. This included Cd, which has been found to exhibit only minor isotope shifts compared with other ocean basins, where greater variations are generated through biological removal [53]. Data from the Swedish-Russian GEOTRACES (GIPY13) cruise to the Siberian shelves found that Cd was not removed in the Lena estuary, and there were further Cd additions to shelf waters from the shelf sediments [54]. Another example of shelf influence on the deep basin is the distribution of Ba, which is strongly enriched in estuarine waters due to desorption from river sediments. In theory, Ba distributions can delineate shelf TEI sources; however, isolating the terrestrial Ba source may be complicated due to biogenic Ba uptake and vertical redistribution [55]. As part of the Canadian IPY-GEOTRACES, a dissolved Ba cross-section through the Canadian Archipelago revealed high surface water Ba concentrations near the Horton River and a pronounced Ba maximum in the upper halocline waters (figure 1; [56]). The latter was thought to be due in part to Ba released to subsurface waters in the wake of organic matter remineralization, a finding similar to Roeske *et al.* [55], who reported that remineralization from the Siberian shelf led to a similar Ba enrichment below the surface mixed layer. This may represent a dynamic process that is not at steady state: such ‘metabolic Ba’ concentrations in the subsurface layer increase with the arrival of organic matter sometime after the spring bloom, approaching maximum values towards the end of winter [56].

**Figure 2.**
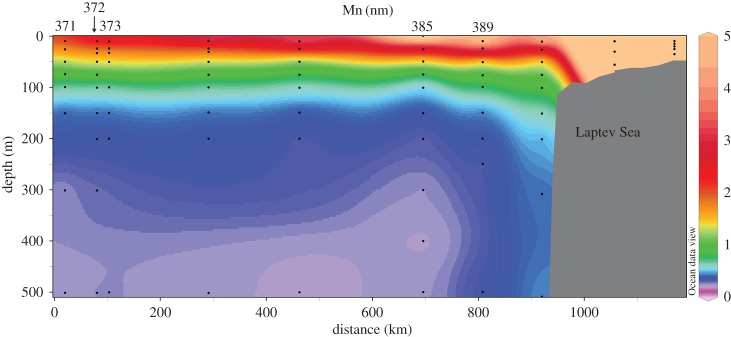
Dissolved Mn (nM) concentrations in the upper 500 m of the Laptev Sea illustrating the strong Mn source over the shelf and its subsequent transport towards the central Arctic basin [57].

A strong Mn enrichment was also found in the surface layer of the central basin due to riverine inputs of Mn (figure 2; [57]), though the inferred river component indicated that river waters were significantly depleted by estuarine processes. Mid-depth enrichments of Mn on the shelf also suggested that there were benthic contributions, though this sediment source did not extend a significant distance off-shelf. The first measurements of Ga in Arctic waters found that its distribution reflected mixing between Atlantic and Pacific waters, with evidence of both riverine input and scavenging removal in shelf waters of the Beaufort Sea [58]. Further studies of the shelf cycling of Ga and related elements (especially Al, which is chemically similar to Ga though more readily scavenged) could provide insights into how shelf scavenging removal affects the off-shelf transport of reactive TEIs.

**Figure 3.**
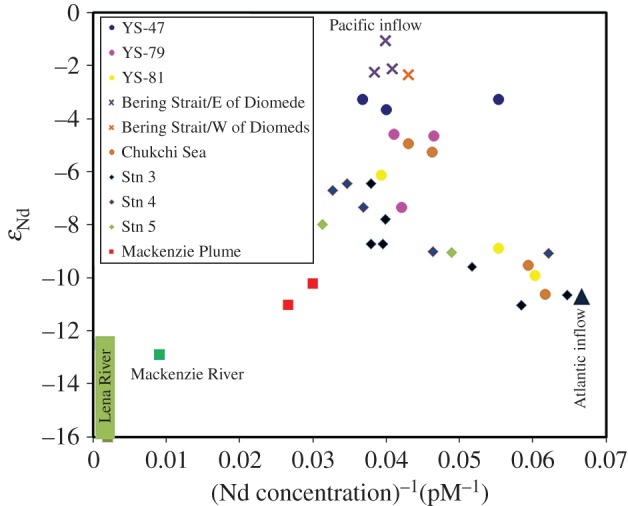
Nd concentration and isotope data for Arctic Ocean waters. The isotope ratios of waters flowing from the Pacific decrease during passage through the Bering Sea before entering the Chukchi Sea in the Arctic due to interaction with shelf sediments [61].

Isotope variations in Nd have been widely used to understand shelf–water interactions and riverine inputs. Within the Arctic Ocean, gradients between surface and halocline waters reflected inputs from the Pacific [59] as well as a source that isotopically matched the major rivers, indicating that the concentrations of the river components reaching the central basin did not reflect the considerable estuarine Nd losses commonly seen elsewhere [60]. These datasets were extended with samples from the BERINGIA 2005 and GIPY13 GEOTRACES cruises, which clearly demonstrated how Nd isotopes and concentrations in the Pacific layer were modified while crossing the Bering Sea through sediment–water exchange processes as was inferred for other shelf areas (figure 3; [61]). Furthermore, Lena River waters did not suffer strong modification through estuarine losses like in the Amazon [62].

Data from GEOTRACES cruises have also documented the behaviour of carbon on the Arctic shelves. Alling *et al.* [63] demonstrated for the first time that substantial degradation of DOC occurs in the Lena River estuary, with greater degradation in the broad East Siberian Seas where shelf water residence times are several years; along with degassing of CO_2_, this process was clearly shown in DIC δ^13^C signatures [64]. Rising Arctic Ocean temperatures are leading to the thawing of permafrost and release of its stored methane [65,66]. Indeed, preliminary results from the recent 2015 US GEOTRACES Arctic section (GN01) show shelf enrichments of tracers such as CH_4_ [67], though the impact of this process on other TEIs remains to be seen. Essential to addressing these and other questions, are radioactive TEIs, which allow for quantification of the time scales associated with these shelf–basin exchange processes, as has been demonstrated by Rutgers van der Loeff *et al.* [68] for ^228^Ra and more recently by Rutgers van der Loeff *et al.* [69], who used the ^228^Th/^228^Ra daughter/parent ratio, which is depleted on the shelves but climbs in the particle-depleted central basin, to estimate an age of 3 years for waters at the Gakkel Ridge.

### (b) The influence of major rivers

**Figure 4.**
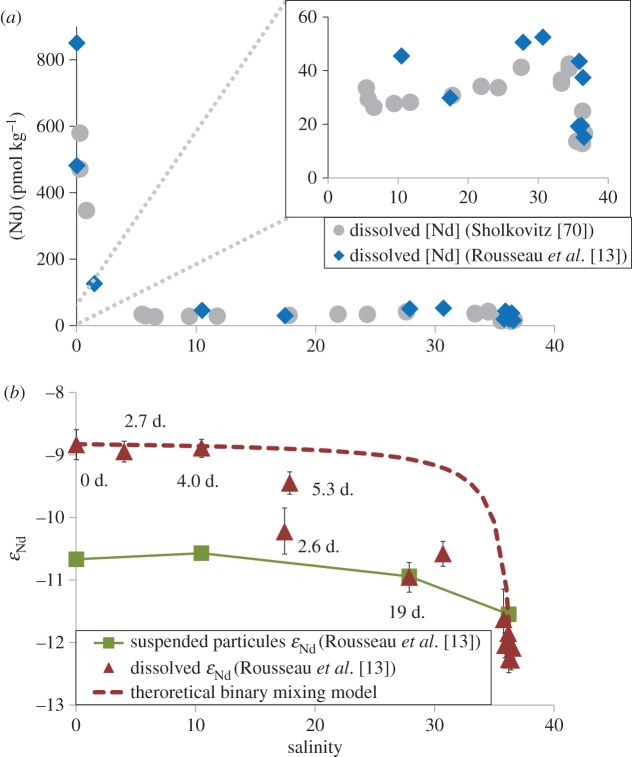
(*a*) Amazon estuary [Nd] from Sholkovitz [70] (circles) and Rousseau *et al.* [13] (diamonds) are reported against the salinity gradient. (*b*) Amazon estuary dissolved (triangles), particulate (squares) 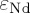 and radium-derived water mass ages (in days) are reported against the salinity gradient. (Online version in colour.)

River-dominated shelves have the potential to be important point sources for TEI delivery to marginal seas and their adjacent ocean basins. For example, Nd isotopic compositions have been measured together with dissolved and colloidal REE concentrations and radium isotope activities in the Amazon estuary salinity gradient as part of the GEOTRACES process study AMANDES (figure 4; [13]). The sharp drop in REE concentrations in the low-salinity region was driven by the coagulation of colloidal material. At mid salinities, dissolved REE concentrations increased, a result of REE release from lithogenic material, a conclusion supported by the Nd isotopic signature within the estuary. Concurrent measurements of the short-lived Ra isotopes (^223^Ra, *t*_1/2_ = 11.4 days and ^224^Ra, *t*_1/2_ = 3.7 days) revealed that this dissolution process is rapid, on the time scale of three weeks. These findings have significant implications for the global marine Nd budget and other TEIs that undergo similar sediment–water exchange processes. This study reinforces one of the original concepts of the GEOTRACES programme: the power of synoptic and multiple TEI sampling approaches to understanding ocean biogeochemical cycling.

### (c) Evidence for eddy-mediated cross-shelf transport of iron

**Figure 5.**
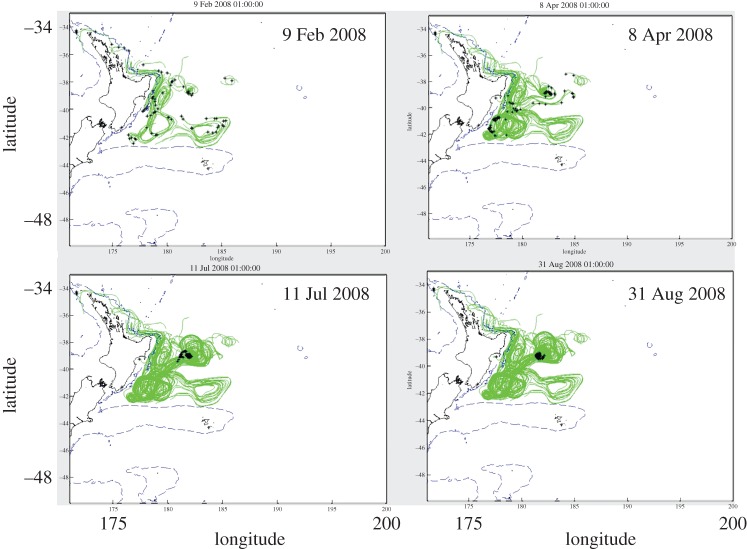
Tracer trajectories (solid lines) from an altimetry model designed to investigate the origin of water masses within a counterclockwise eddy studied as part of the GEOTRACES FeCycle II process study [78]. Model snapshots are from (clockwise starting at top left) 9 Feb, 8 April, 11 July and 31 Aug 2008. The tracers (black symbols) traverse the waters on and across the 200 m deep shelf break (dashed contour lines) adjacent to the eastern seaboard of the northern island of New Zealand. (Online version in colour.)

Although dust deposition is often considered the dominant external source of iron to the open ocean, it has now been well established that long-range transport of shelf Fe in high-nutrient low-chlorophyll (HNLC) regions are a factor in the development of blooms 100s–1000s of kilometres offshore (e.g. [37,71–73]) and can dominate iron supply on the global scale [74]. While radium isotopes have been used to quantify this source [75–77], isolating the shelf source on basin-scales is not easily accomplished in regions beyond the Southern Ocean where other inputs (e.g. dust, hydrothermal vents) may be co-occurring. A 2008 GEOTRACES process study, ‘FeCycle II’, focused on biogeochemical cycling within an eddy off the eastern seaboard of the north island of New Zealand, which is seasonally oligotrophic following the spring diatom bloom [78]. The study revealed that the iron supply for these blooms comes from cross-shelf transport of metals that are likely ‘picked up’ on the shelf and moved offshore as an eddy is formed. This conclusion was reached based on high dissolved and particulate Mn within the eddy and from trajectory analysis using a satellite altimetry model (figure 5).

### (d) Apportioning sources of iron using iron isotopes

**Figure 6.**
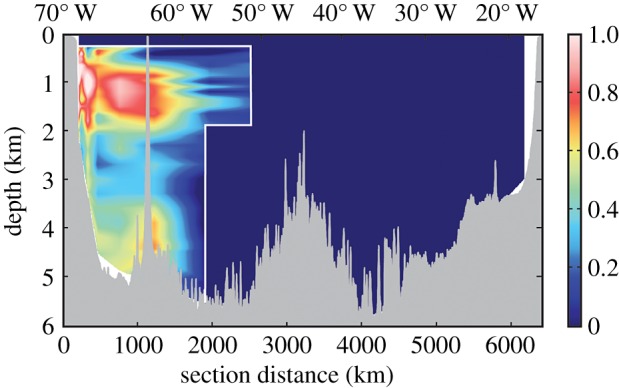
Fraction of water column Fe associated with input from oxygenated sediments along the North Atlantic margin (from [84]).

In addition to transport models, isotopes of iron have recently been used as tracers of oceanic Fe sources [79–82]. Novel high-throughput methods [83] have enabled high-resolution sampling on ocean section cruises like GEOTRACES. Recently, Conway & John [84] used this approach to apportion iron sources to the North Atlantic according to dust input, hydrothermal venting and two types of sediment fluxes: reductive and non-reductive sedimentary release. While they estimated that dust was the dominant Fe source, they reported that non-reductive release from sediments on the North American margin was a major local source that contributed between 10 and 19% of the iron basin-wide (figure 6). In addition, Fitzsimmons *et al.* [85] reported that approximately 60–80% of the dissolved Fe in this region was in the colloidal phase, which has implications for the bioavailability and long-range transport of this important micronutrient. At the African margin, reductive dissolution in sediments accounted for 1–4% of the iron basin-wide [84]. Further south, Homoky *et al.* [86] attributed a high-proportion of dissolved Fe present in margin sediments to non-reductive release, and earlier studies of pore waters that were rich in colloidal iron had similar isotope compositions [87,88], which supports the view that colloids may influence the stability and transport of iron from non-reductive sediment sources in ocean basins [89].

### (e) Time variations in basin-scale submarine groundwater discharge

Submarine groundwater discharge has received increased attention over the past two decades as a source of TEIs to the ocean. The majority of the early studies focused on the local scale, though Moore *et al.* [24] was able to estimate SGD to the Atlantic Ocean using ^228^Ra (*T*_1/2_ = 5.75 yr) inventories from the Transient Tracers in the Ocean (TTO) programme, and determined that the SGD flux was 2–4 × 10^13^ m^3^ yr^−1^, equivalent to 80–160% of the freshwater discharge from rivers. Since the TTO data had been collected in the 1980s, the Atlantic Ocean ^228^Ra inventory had largely decayed and been replaced by the time of the 2010–2011 US GEOTRACES North Atlantic programme. This afforded Charette *et al.* [90] the opportunity to evaluate whether or not this ocean basin was in steady state with respect to SGD inputs. Using ^228^Ra data collected along transects between North America and West Africa, and Western Europe and West Africa, they observed essentially no change in the upper ocean inventory of this tracer, suggesting that SGD had not changed despite significant changes in groundwater withdrawals during the intervening period.

Kwon *et al.* [26] took this a step further and used inverse modelling techniques applied to a global ^228^Ra dataset to calculate total SGD to the ocean. This approach yields the total ^228^Ra flux from the shelf, which in addition to the SGD input includes the riverine discharge and shelf sediment diffusive sources. Sediments of continental shelves and aquifers are important areas for *in situ* production of Ra isotopes through continuous decay of their parent thorium isotopes (e.g. [91]), while rivers supply dissolved Ra isotopes as well as Ra sourced from desorption from suspended sediments in the estuarine mixing zone [92]. For a number of TEIs, estimates for riverine inputs are generally well constrained, however, due to estuarine processing and direct TEI inputs to the shelf we lack a method or approach for quantifying the net flux of TEIs across the interface between coastal and open ocean waters.

### (f) ^228^Ra as a shelf trace element and isotope flux gauge

To this end, we are proposing an approach for quantifying shelf TEI fluxes that uses ^228^Ra as a shelf flux gauge. This method takes advantage of the global inverse model of Kwon *et al.* [26], which focused on isolating the flux ^228^Ra via SGD to the ocean, but at its root is designed to estimate the total ^228^Ra flux from all shelf sources required to balance the upper ocean ^228^Ra inventory and decay. Because of its strong shelf source and relatively short half-life (on the time scale of mixing), the majority of the upper 1000 m ^228^Ra inventory in the basin can be traced back to the shelf. This inverse approach to estimating shelf ^228^Ra flux has the advantage of integrating the shelf source of ^228^Ra over annual to decadal timescales, which averages out seasonal variability that hampers the use of near shore ^228^Ra gradients to estimate shelf ^228^Ra fluxes directly [93]. As a first-order estimate, we propose to use the ratio of near shore gradients of dissolved TEI and ^228^Ra measured over the shelf and nearby stations during specific GEOTRACES cruises to link the model-derived shelf–ocean ^228^Ra flux to shelf–ocean TEI fluxes.

**Figure 7.**
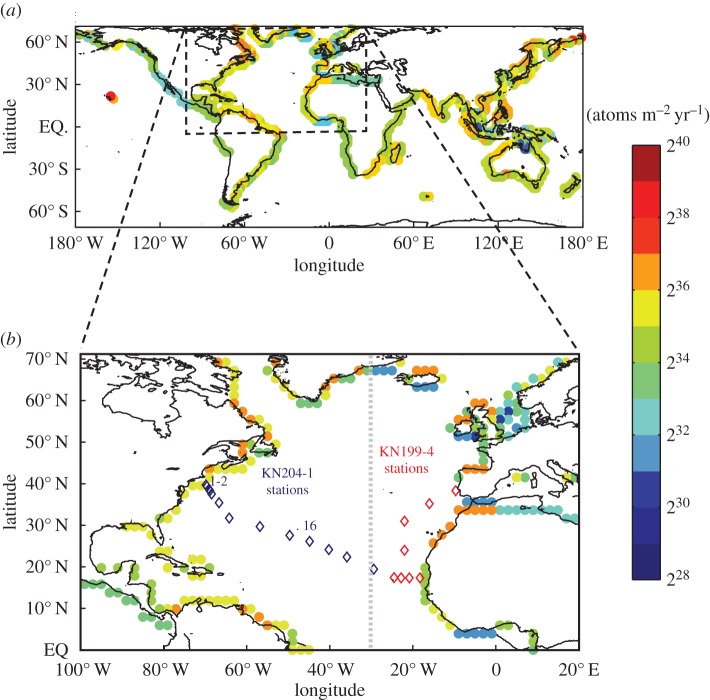
(*a*) Model derived shelf ^228^Ra flux (units are log base (2) atoms m^–2^ yr^–1^) from the model of Kwon *et al.* [26]. Also shown in (*b*) are the US GEOTRACES GA03 cruise stations (diamonds). The dashed line in (*b*) is the boundary between the eastern and western Atlantic margins. The innermost coastal and central Atlantic stations were used to derive the ΔTEI/Δ228Ra averages.

The full details of the global ^228^Ra model can be found in Kwon *et al.* [26]. Briefly, the model employs a 2° × 2° global circulation model where the domain is restricted to between 60° S and 70° N due to insufficient ^228^Ra coverage in the polar oceans. The vertical resolution is fine near the surface (approx. 40 m) and coarse near the ocean bottom (approx. 600 m). The coastal ^228^Ra source is defined as that originating from the ocean grid boxes adjacent to land boxes with a depth of approximately less than 200 m. The coastal source is optimized through a minimization scheme whereby the reported fluxes are those that result in the best fit between the model and observed ^228^Ra activities in the basin. The total ^228^Ra fluxes for each 2° × 2° margin grid cell are shown in figure 7*a*. The highest total margin inputs are to the North Pacific and Indian Ocean basins. For both the Atlantic and Pacific Oceans, the western margin ^228^Ra fluxes exceed those from the east, probably due to a combination of major river inputs, SGD, and the presence of broad continental margins and/or extensive shelf seas. The relatively narrow shelf along the North American active margin in the Pacific appears to have the lowest inputs on average.

Assuming shelf–ocean exchange is primarily driven by eddy diffusion, the net cross-shelf TEI flux can be linearly scaled with the net cross-shelf ^228^Ra flux as follows:
2.1
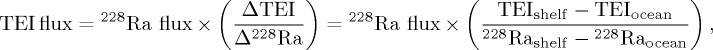

where TEI_shelf_ and ^228^Ra_shelf_ are the average concentrations of the TEI of interest and ^228^Ra over the shelf water column (less than 200 m). The TEI_ocean_ and ^228^Ra_ocean_ are the average dissolved TEI and ^228^Ra in the open ocean (less than 200 m; see the electronic supplementary material). For highly reactive elements with very low open ocean concentrations, this ratio may be close to (TEI_shelf_ /^228^Ra_shelf_). However, for this approach to be applicable to TEIs with a wide range of particle reactivities, including those with non-negligible open ocean concentrations relative to shelf concentrations, ΔTEI/Δ^228^Ra should be employed. For shelves where the net cross-shelf advective flux is substantial, the TEI flux would not scale linearly with ^228^Ra flux as illustrated in the electronic supplementary materials.

It is important to recognize that fluxes derived from this approach are the net dissolved TEI input rate to the ocean at the shelf break (200 m). Hence, the flux at this boundary is not necessarily what might be expected to reach the ocean interior due to the varying degrees of TEI particle reactivity and biological cycling. Further, the method in theory should account for any TEI removal over the shelf; therefore, fluxes may not equal the sum of the inputs along the boundary (e.g. rivers, SGD, sediment diffusion). Finally, we note that many of the TEI shelf input and removal processes vary seasonally, not necessarily in concert with seasonal variability in ^228^Ra sources, and that not all shelf sources are expected to have uniform ΔTEI/Δ^228^Ra. For example, sporadic sources due to rivers and SGD may hinder a proper averaging of ΔTEI/Δ^228^Ra over large shelf areas. While the spatial and temporal variability in a particular ΔTEI/Δ^228^Ra must be fully assessed before this method is to be widely employed, we hope that this exercise provides a first-order assessment of the importance of shelf TEI fluxes to the ocean in comparison to other external sources.

For the purpose of this exercise, we chose to focus on the North Atlantic Ocean basin due to the availability of synoptic TEI and ^228^Ra data from the US GEOTRACES GA03 cruises, though the scope could be expanded as more GEOTRACES datasets become available. These cruises crossed or approached three main shelf areas: the northwest Atlantic shelf south of Woods Hole, MA (USA), the Iberian margin and the Mauritanian upwelling zone off of western Africa. For perspective, the combined North Atlantic shelf ^228^Ra flux (23.9 ± 4.6 × 10^22^ atoms yr^−1^) accounts for approximately 25% of the global shelf flux (96 ± 5 × 10^22^ atoms yr^−1^; figure 7*a*; [26]). Of the three GA03 cruise shelf crossings, however, only the northwest Atlantic has multiple stations in close proximity to the shelf break and a shelf where elemental transport is dominated by eddy diffusion [94]. As a result, the western North Atlantic shelves (0°–70° N), which are responsible for about 60% of the shelf ^228^Ra input to this ocean basin (14.3 ± 1.9 × 10^22^ atoms yr^−1^), will be the focus of our shelf TEI flux calculations.

Though there is a long list of TEIs fluxes that could be determined using this method, we chose to focus on four (dissolved Fe, Mn, Co, Zn) that span a range of particle reactivity and play a role in upper ocean biogeochemical cycling. The ΔTEI/Δ^228^Ra ratios were calculated using equation (2.1) from averaged concentration data for the two northwest Atlantic near shore stations (GA03, KN204–1 stations 1,2) and open ocean station 16 (GA03, KN204–1; figure 7*b*).

By combining the model ^228^Ra fluxes and ΔTEI/Δ^228^Ra, we can estimate the annual shelf TEI inputs to the western North Atlantic Ocean (table 1). The western North Atlantic shelf Co flux (1.4 ± 0.4 × 10^8^ mol yr^−1^) is consistent with the literature estimates from a variety of independent approaches. Saito *et al.* [95] estimated that the shelf dissolved Co flux for the Peru upwelling region was 2.0 × 10^7^ mol yr^−1^, which compares well with our estimate considering that we integrated over an approximate seven times larger area. Lateral shelf area normalized Co fluxes of 6.2–10 µmol m^−2^ yr^−1^ were reported by Bown *et al.* [96] for the South Atlantic near Cape Town. These are a factor of approximately 5–10 lower than the shelf-normalized fluxes for the western North Atlantic margin (table 1; 56 µmol m^−2^ yr^−1^), though their estimate was based on transport across a boundary several hundred kilometres from the shelf break.

**Table 1.** Western North Atlantic Ocean margin TEI flux estimates derived from shelf ^228^Ra inputs (14.3 ± 1.9 × 10^22^ atoms yr^−1^; 0–70° N) and ΔTEI/Δ^228^Ra ratios. The integrated shelf area used to normalize the basin-scale fluxes was 2.5 × 10^12^ m^2^.

	dCo	dFe	dMn	dZn
TEI/^228^Ra (× 10^−6^ nmol atom^−1^)	1.0	2.7	3.8	11
TEI flux (× 10^8^ mol yr^−1^)	1.4	3.9	5.4	16
TEI flux (µmol m^−2^ yr^−1^)	56	160	220	630

The ΔTEI/Δ^228^Ra approach yielded a shelf Fe flux of 3.9 ± 1.4 × 10^8^ mol yr^−1^ for the western North Atlantic. When normalized to shelf area, this flux is 160 µmol m^−2^ yr^−1^. Sedimentary Fe inputs [89], which are expectedly higher as they do not account for any removal over the shelf, range from 900 [74] to 1570 [72] to 2700 µmol m^−2^ yr^−1^ [97]. On a global scale, the shelf-sedimentary Fe inputs as reported by Tagliabue *et al.* [74], Elrod *et al.* [72] and Dale *et al.* [97] are 2.7 × 10^10^, 8.9 × 10^10^ and 7.2 × 10^10^ mol yr^−1^, respectively. The western North Atlantic Ocean total shelf input as determined by our method would therefore represent only 0.4–1.4% of the global sediment flux. If we assume that our ΔFe/Δ^228^Ra is comparable to the global shelf average, our approach would predict a global shelf–ocean Fe flux of 2.3 × 10^9^ mol yr^−1^. If the western North Atlantic shelf is representative of shelf systems globally, our model suggests that only a small fraction of the shelf-sedimentary Fe input is exported to the open ocean and therefore available for biological uptake where Fe may be limiting.

The western North Atlantic Mn shelf flux is 5.4 ± 1.0 × 10^8^ mol yr^−1^ or 220 µmol m^−2^ yr^−1^. The literature values for shelf Mn fluxes are largely focused on the shelf sediment source. For example, Landing & Bruland [98] reported sedimentary Mn flux of up to 140 µmol m^−2^ yr^−1^ for the Monterey shelf, while McManus *et al.* [99] observed much higher values for the Oregon/California shelf (2900 ± 900 µmol m^−2^ yr^−1^). The former agrees quite well with our estimate based on equation (2.1), whereas the latter is likely to be higher due to the high productivity associated with the strong upwelling in that region. Lastly, the total Zn shelf flux is 1.6 ± 0.6 × 10^9^ mol yr^−1^ or 630 µmol m^−2^ yr^−1^. To the best of our knowledge, the shelf Zn flux estimates reported herein are the first of their kind.

In terms of other major sources to the surface ocean, shelf inputs can be on par with or even dominant for certain TEIs. The dissolved cobalt flux for the western North Atlantic shelf alone is over an order of magnitude higher than the atmospheric deposition of soluble Co to the entire ocean basin as reported by two independent studies (approx. 11 × 10^6^ mol yr^−1^; [100,101]). Soluble Fe (wet + dry) atmospheric deposition to the tropical North Atlantic ranges from 2.9–43 µmol m^−2^ yr^−1^ [102]; scaled to the basin the atmospheric Fe flux becomes 1.2–18 × 10^8 ^mol yr^−1^ or 31–460% of the western North Atlantic dissolved shelf flux using the TEI/^228^Ra approach. Powell *et al.* [102] also reported soluble (wet + dry) atmospheric Mn fluxes, which we scaled to the North Atlantic (0.75–15 × 10^8^ mol yr^−1^), equivalent to 14–280% of the shelf inputs reported herein. Assuming 15% solubility, Little *et al.* [103] estimated the atmospheric Zn input to the surface ocean to be 6.9 × 10^7^ mol yr^−1^; our estimates for the western North Atlantic shelf alone exceed that flux by a factor of approximately 23. Higher concentrations of Zn along with lighter isotopes were observed at both eastern and western Atlantic margins indicating sediments were a source of Zn to this region [84]. Our net shelf–ocean flux of Zn is almost a factor of three higher than the Little *et al.* [103] *global* estimate for riverine input (5.9 × 10^8^ mol yr^−1^); this is in contrast with their suggestion that scavenging removal of Zn and burial in continental margin sediments might represent the ‘missing sink’ for Zn in the global ocean mass balance for this element.

## 3. Recommendations for the future

We have presented a possible path forward in quantifying TEI shelf–open ocean exchange rates using ^228^Ra and demonstrated the potential of the method by focusing on the western North Atlantic Ocean. This exercise was made possible by publication of a recent global model for shelf radium inputs and synoptic TEI and ^228^Ra measurements on a series of US GEOTRACES cruises in 2010–2011. Since Ra isotope measurements are not a requirement for GEOTRACES compliance, we suggest that future section cruises and shelf process studies include at least ^228^Ra so that we can better understand how to relate this tracer to other TEIs. Ra isotope data are especially needed for the Indian and Pacific Oceans where historical data coverage is sparse. Shelf process studies would be needed for a range of shelf settings, i.e. how do ΔTEI/Δ^228^Ra ratios vary seasonally and as a function of hydrological state, shelf width and coastline lithology (e.g. karst versus volcanic)? Lastly, for shelf environments where advection plays an important role in TEI transport, a second conservative tracer in addition to ^228^Ra would be needed to constrain the shelf–ocean TEI flux (electronic supplementary material).

While we have used an inverse approach, which was based on a coarse resolution model, in order to calculate shelf fluxes at a near basin-wide scale, a finer resolution model needs to be combined with coastal ^228^Ra and TEI data in order to constrain various shelf TEI sources more precisely. Where ^228^Ra measurements are not possible on future GEOTRACES cruises, we advocate for concurrent physical measurements that may also be used to quantify the shelf flux of TEIs. For example, Tanaka *et al.* [104] combined DFe distributions with turbulence measurements using a vertical microstructure profiler (VMP) in the Bering Sea; they found that productivity in this region was driven in part by injections of iron-rich subsurface layer at the southeastern shelf break.

Our discussion above highlights the potential importance of shelf processes on open ocean TEI distributions. Results to date are somewhat limited because of the programmatic emphasis placed on open ocean full-depth profiles. For example, lack of data over the shelf for GA03 precluded the inclusion of the eastern boundary shelves in our analysis of TEI fluxes to the North Atlantic Ocean. To better understand the role of shelf input to the open ocean (and vice versa) in global TEI budgets, future GEOTRACES sections may need to be reconfigured with an increased emphasis on shelf stations. Given the shallow depths involved, this change would not impact ship-time requirements to any significant extent. Also, sections in regions with wide shelves and high ratios of shelf area to open water will be particularly useful. The recent 2015 Canadian, US and German sections in the Arctic Ocean are examples of this approach. Fortunately, Ra isotopes were measured on all three cruises.

There are a number of margin-centric GEOTRACES sections that have been identified in the programme planning documents but have yet to be realized due to a variety of factors. These include two of the three proposed for the coastal China seas, Brazil margin and the Gulf of Mexico. Regarding the latter, the 2007 GEOTRACES Atlantic Workshop Report identified a section through the Caribbean and Gulf of Mexico that contains significant opportunities to examine shelf impacts. Roughly, one-third of the area of the Gulf of Mexico comprises shelf waters less than 200 m deep. Portions of the coastline are river-dominated (Mississippi), whereas others are groundwater run-off-dominated carbonate platforms (Yucatan peninsula, southern Florida). Furthermore, the Loop Current, a major oceanic current, runs through the Gulf, variably interacting with the shelf. Thus, the Gulf of Mexico is a unique basin for the study of margin–open ocean interactions. Surprisingly, though, despite the significant interest in Louisiana Shelf hypoxia in the northern Gulf as well as recent studies engendered by the Deepwater Horizon blowout, few studies have addressed the issue of the shelf's influence on open Gulf waters and then generally only in a tangential way. For instance, early studies by Brooks *et al.* [105], Reid [106] and Todd *et al.* [107] all pointed to the likelihood of off-shelf transport of methane and radium in the Gulf. Likewise, Trefry & Presley [108] suggested that Mn fluxes from shelf sediments provided a source for ‘excess’ Mn in deep Gulf of Mexico sediments. Nonetheless, these studies have not been followed up by more detailed surveys or process studies. Surprisingly, TEI distributions in open waters of the Gulf are generally unknown.

In this report, we have summarized evidence supporting the importance of continental shelves and shelf seas in the oceanic mass balance of TEIs. Furthermore, we have outlined a methodology using ^228^Ra to more consistently estimate the flux of TEIs from the margins to the open ocean. To improve these estimates, we recommend that GEOTRACES sections place more emphasis on sampling along the margins and that increased consideration be given to completing margin-focused sections, such as that previously proposed for the Gulf of Mexico.

## Supplementary Material

Supplementary Material

## Data accessibility

For data supporting this article, see http://data.bco-dmo.org/jg/dir/BCO/GEOTRACES/NorthAtlanticTransect/.
