# Supplementary material for: Coastal ocean and shelf-sea biogeochemical cycling of trace elements and isotopes: lessons learned from GEOTRACES
Source: Philos Trans A Math Phys Eng Sci. 2016 Nov 28;374(2081):20160076. doi: 10.1098/rsta.2016.0076 (PMC5069537; doi:10.1098/rsta.2016.0076)
Supplement: Supplementary Material [file rsta20160076supp1.docx]

**Supplementary Material**

We describe the method that allows us to calculate the net cross-shelf TEI flux based on the net cross-shelf ^228^Ra flux. Consider an ocean domain consisting of three boxes: Box 1 is the shelf shallower than 200m, Box 2 is the adjacent open ocean shallower than 200m, and Box 3 is the rest of the ocean including most of the open ocean and the deep ocean (Supplementary Fig. 1). Box 3 could also be adjacent shelf areas that are not included in Box 1. Note that the shelf box (Box 1) includes part of the grids adjacent to the land grids in a horizontal resolution of 2°x2° (~ 200 km x 200 km).

**Supplementary Figure 1.** Three box model used to conceptualize the derivation of ΔTEI/Δ^228^Ra ratios for different shelf-ocean exchange processes.

The volumes of the boxes are V_1_, V_2_, and V_3_. There are two tracers, R and D, representing ^228^Ra and the TEI of interest, respectively, each with units of mol/m^3^. The concentrations of the tracers in each box can be denoted as R_1_, R_2_, R_3_ and D_1_, D_2_, and D_3_. The advective flux of water from Box 1 to Box 2, from Box 2 to Box 3, and from Box 3 to Box 1 is T, and the two-way diffusive flux of water between Box 1 and Box 2 is f_12_, between Box 2 and Box 3 is f_23_, and between Box 3 and Box 1 is f_13_. The units for T and f are m^3^/y.

In this example, the governing equations for R_1_, R_2_, and R_3_ are:

$V_{1}\frac{\partial R_{1}}{\partial t}={Flux}_{3to1}(R)-{Flux}_{1to2}(R)-V_{1}\frac{ln2}{5.75 y}R_{1}+V_{1}S$ (S1)

$V_{2}\frac{\partial R_{2}}{\partial t}={Flux}_{1to2}(R)-{Flux}_{2to3}(R)-V_{2}\frac{ln2}{5.75 y}R_{2}$ (S2)

$V_{3}\frac{\partial R_{3}}{\partial t}={Flux}_{2to3}(R)-{Flux}_{3to1}(R)-V_{3}\frac{ln2}{5.75 y}R_{3}$ (S3)

where *ln2/5.75y* is the decay constant for ^228^Ra in 1/y and *S* is the coastal source of ^228^Ra due to SGD, rivers and coastal sediments in mol/m^3^/yr . The ${Flux}_{1to2}\left( R \right)$, for example, denotes the net flux of R from Box 1 to Box 2 in units of mol/y. The ${Flux}_{1to2}(R)$consists of both advective and diffusive fluxes:

${Flux}_{1to2}\left( R \right)=TR_{1}+f_{12}\left( R_{1}-R_{2} \right).$ (S4)

If we assume a steady-state, and sum up equations (S1) - (S3), then we obtain:

$\frac{ln2}{5.75 y}{(V_{1}R}_{1}+{V_{2}R}_{2}+{V_{3}R}_{3})=V_{1}S,$ (S5)

such that the volume integrated coastal source *V_1_S* balances the total sink of R due to radioactive decay .

Note that the decay term for ^228^Ra, $\frac{ln2}{5.75 y}R_{1}$ is very small . If we further assume that ${Flux}_{3to1}(R)$ is negligible compared to ${Flux}_{1to2}\left( R \right), from equation S\left( 1 \right), we obtain$

${Flux}_{1to2}\left( R \right)=TR_{1}+f_{12}\left( R_{1}-R_{2} \right)\approx V_{1}S.$ (S6)

Likewise, the net D flux from Box 1 to Box 2 can be written as:

${Flux}_{1to2}\left( D \right)=TD_{1}+f_{12}\left( D_{1}-D_{2} \right).$ (S7)

If advective transport can be considered negligible, which would be the case for large scale Northwest Atlantic coastal regions [1] , then the advective fluxes TR_1_ and TD_1_ can be dropped from equations (S6) and (S7). In this case equation (S7) can be combined with equation (S6) and re-written as

${Flux}_{1to2}\left( D \right)\approx f_{12}\left( D_{1}-D_{2} \right)={Flux}_{1to2}\left( R \right)\times\frac{\left( D_{1}-D_{2} \right)}{\left( R_{1}-R_{2} \right)}=V_{1}S\times\frac{\left( D_{1}-D_{2} \right)}{\left( R_{1}-R_{2} \right)}$ (S8)

which is the approach we have taken in the paper for the western North Atlantic margin.

If net advective fluxes are not negligible, then both the advection and eddy mixing terms in equations (S6) and (S7) are important for determining the shelf TEI flux. In this case, we cannot scale the flux of one tracer in terms of the other. Since we have two unknowns, R and f_12_, we need at least one more equation along with equation (S6). The additional equation can be obtained from a governing equation for another tracer, whose sources and sinks are relatively well known. Once we calibrate f_12_ and T, we can use equation (S7) to diagnose the TEI flux.

**Literature Cited**

1. Moore WS. Determining coastal mixing rates using radium isotopes. Cont Shelf Res. 2000;20(15):1993-2007.
